# Hypnosis in a specialist palliative care setting – enhancing personalized care for difficult symptoms and situations

**DOI:** 10.1177/2632352420953436

**Published:** 2020-10-12

**Authors:** Sara Booth

**Affiliations:** Hon Consultant Palliative Care Service, Cambridge University Hospitals NHS Foundation Trust (CUHNHSFT), Cambridge, UK; Hon Sen Lecturer, Cicely Saunders Institute, King’s College London, London CB2 0QQ, UK

**Keywords:** cancer, distress, hypnosis, intervention, non-malignant disease, research, self-training, specialist palliative care, supportive care, symptom

## Abstract

This is a personal account of using hypnosis as an adjunct to specialist palliative care (SPC) treatment approaches. After a brief systematic review of the literature, one clinician’s experience is outlined illustrated by short, anonymized case histories. It argues that the approach is underused in SPC. The barriers currently restricting its routine adoption in SPC are discussed including (1) a lack of SPC clinical trials, (2) a misunderstanding of hypnosis leading to stigma, and (3) its absence from clinicians’ training pathways. While the evidence base for the effectiveness of hypnosis in ‘supportive care’, for example, managing chemotherapy-induced vomiting, is appreciable, there is a gap in SPC. There is little data to guide the use of hypnosis in the intractable symptoms of the dying, for example, breathlessness or the distress associated with missed or late diagnosis. There are many people now ‘living with and beyond cancer’ with chronic symptomatic illness, ‘treatable but not curable’. Patients often live with symptoms over a long period, which are only partially responsive to pharmacological and other therapies. Hypnosis may help improve symptom control and quality of life. SPC trials are needed so that this useful tool for self-management of difficult symptoms can be more widely adopted.

## Background

Specialist palliative care (SPC) services care for patients (and their families) with incurable illness, most approaching the last months or weeks of their life. Many people experience continuing psychological and physical symptoms, even after maximal treatment of their underlying disease, and frequently, these are only partially responsive to pharmacological and other current therapies. It is a fundamental to SPC that symptoms are complex experiences involving psychological, spiritual, and social aspects that need a range of treatments focusing on the needs and priorities of the patient, rather than following fixed treatment algorithms.^1^

There has been a growing appreciation of the limitations of opioids and other drug therapy in the control of chronic symptoms like neuropathic pain, during the end-of-life (EOL) phase and in chronic illness. This has led to calls for increased investigation of non-pharmacological and self-management approaches to symptom control in all settings.^2,3^

Hypnosis seems an ideal intervention for SPC, given the need for additional approaches to symptom control and the possibility of enhancing the individual’s ability to implement self-management using self-hypnosis. There are few interventions as personalized, nor with such a wide therapeutic potential to manage an individual’s symptoms^4^ or exacerbators of symptoms. Hypnosis is portable, can be used in any setting, has no adverse effects and, once learned, is very economical.^5^ It is led by the individual and can be reshaped to meet new priorities and symptoms. It can help with both psychological and physical difficulties and indeed, the approach recognizes that these are not easily divisible.

Nonetheless in spite of its potential advantages, hypnosis is not widely used in SPC. This may partly be due to its unfamiliarity: it is not is routinely introduced as a therapeutic option in medical schools or on specialist training programmes, even in specialties where a high proportion of patients live with difficult symptoms. It may also be because it is misunderstood and stigmatized by its unfortunate associations in the lay media with showbusiness.^6^ Hypnosis is often presented here as an interaction where the client hands over control of their mind to another powerful individual, who makes them carry out acts antithetical to their belief system and normal behaviour. In addition, the excellent evidence for its effectiveness in, for example, irritable bowel syndrome^7^ is not widely known. There is no good-quality evidence specific for SPC.

This article gives an overview of the academic basis for hypnosis and an introduction to the practicalities of integrating the technique into routine SPC clinical practice, alongside other treatments. It includes a summary of the results of a short systematic review of the current SPC evidence and refers to that available in supportive cancer care. A personal account of using hypnosis in SPC follows with brief anonymized case studies. The importance of paying attention to ‘waking suggestion,’ that is, the language used in everyday therapeutic encounters is highlighted. Ann Williamson has outlined current ideas on mechanisms for hypnosis in a recent issue^8^ of this journal and these are not repeated here.

## Definitions of hypnosis

Kirsch defined hypnosis as

‘a procedure during which a health professional or researcher suggests that a client, patient, or subject experience changes in sensations, perceptions, thoughts, or behaviour. The hypnotic context is generally established by an induction procedure. Although there are many different hypnotic inductions, most include suggestions for relaxation, calmness, and well-being. Instructions to imagine or think about pleasant experiences are also commonly included in hypnotic inductions.’^9^

In essence, hypnosis encompasses a state of intense inner focus, in which the client is less aware of external distractions and can be induced by a clinician or therapist or an individual can learn to induce a hypnotic state in themselves, usually called ‘self-hypnosis’. More recently, the American Psychological Association (APA) came to a consensus about a new definition ‘a state of consciousness involving focused attention and reduced peripheral awareness characterized by an enhanced capacity for response to suggestion’^10^ which encompasses self-hypnosis. It was felt that any definition needed to be ‘concise’ as well as ‘heuristic’ to allow ‘alternative theories of mechanism’. There are many definitions of hypnosis and the lack of an agreed one is a barrier to research.

## How is hypnosis currently used in palliative care and cancer medicine by clinicians?

Hypnosis is used as an adjunct to, and augmentation of, other treatments in clinical practice (see Box 1 for possible indications in supportive and palliative care).

**Box 1.** Possible Applications of hypnosis in palliative and supportive care.

**Possible Applications of hypnosis in palliative and supportive care** **Symptom control**, e.g., nausea & vomiting, pain, breathlessness, and poor sleep **Managing adverse effects of or fears about treatment**, e.g., anticipatory vomiting from chemotherapy, needlephobia, mask phobia in head & neck cancer, and fear of enclosure in MRI **Reducing medication needs during procedures**, e.g., adjunct or replacement to sedation during gastroscopies, colonoscopies, and minor surgery **Supporting self-management of difficult situations or feelings**, e.g., distress, stress, anxiety, fear, panic, and managing difficult personal relationships or memories **Improving general health**, e.g., supporting exercise, eating habits, and sleep hygiene/quality

It can enhance the use of medical advice, as well as pharmacological and non-pharmacological interventions. In clinical practice, hypnosis is not regarded as a treatment in itself and many avoid the term hypnotherapy for this reason. As Williamson^8^ points out, the state of being deeply relaxed is recognized as helpful for reducing the impact of stress, and most SPC hypnosis sessions include a focus on relaxation. Like other clinical interventions, the setting in which hypnosis takes place, the disposition of the therapist, and their ability to develop a therapeutic relationship with the patient and the wider context of its use will contribute to any useful additional placebo effect gained from the therapeutic rituals of palliative care consultations.^11^ The context may include the atmosphere in the hospice, the time given to patient and family, the status of the clinician as experienced in palliative care and hypnosis and their confidence in it. With difficult symptoms and situations, it is particularly important to harness any possible therapeutic gain.^12^

There are a range of models that clinicians may use to explain hypnosis, outlined in Williamson’s paper.^8^ Explaining that hypnotic phenomena are part of everyday experience may be helpful, for example, being engrossed in a book and failing to notice someone coming into the room.^13^ Further discussion of these issues may be found in standard texts^5,14^ or on the websites of learned societies, for example, the British Society of Clinical and Academic Hypnosis (BSCAH), which includes a section on explaining hypnosis to patients (https://www.bscah.com/#home).

## The impact of cancer and other serious illnesses

Cancer is a multi-system disorder with many physical and psychological symptoms, many of which may be difficult to manage. Treatment often has adverse effects. Most adult cancers fall into the category of ‘treatable but not curable’, so the patient may need support living with a chronic illness or facing a rapidly fatal one. The diagnosis has profound psychosocial impacts – causing significant ‘biographical disruption’.^15^ There are often important losses associated with the diagnosis, employment changing, a business folding, some relationships may end, and an individual may lose their strong sense of a previous identity. There will often be financial pressures. For some, it prompts difficult existential questions, for others, a loss of, or a new adherence to a religion. Family relationships can shift helpfully or in a disruptive, painful way. Even though other conditions may have even more serious physical consequences than some easily treatable cancers, the disease is still regarded as the ‘iconic’ illness^16^ evoking an emotional response in family, friends, and casual acquaintances. These reactions can be a problem for the sufferer^17,18^ but have also lead to greater investment in cancer services, including supportive care, than in other diseases with comparable prognoses.

The issues of living ‘with and beyond cancer’ (previously called ‘survivorship’) are becoming recognized and systems developed to meet these needs but it is clear that many people are still feeling ill-equipped to manage the long-term sequelae of the illness^19,20^ such as fluctuating anxiety or fatigue. This experience of ‘treatable, not curable’ is the experience of many people with non-malignant disease so that the trajectories of some people with cancer and others with non-malignant disease are becoming more similar, and palliative care services now aim to offer care based on ‘needs not diagnosis’.

## The limitations of pharmacological symptom control

Symptom control in advanced diseases like cancer and chronic obstructive pulmonary disease (COPD), such as neuropathic pain, poor sleep, fatigue, or breathlessness, is often only partially effective and patients continue to live with considerable suffering, which also affects their carers.^21^ Out-of-hours use of medical services, when there is less clinical support, is associated with uncontrolled symptoms^22^ and associated anxiety. This is often futile, as there is no apparent clinical change and adds to patients’ burdens. Self-hypnosis training targeting difficult symptoms will give the patient an independent clinical approach. Pharmacological treatment will not help feelings of loss of meaning, regret, and emotional distress – hypnosis may enable people to gain a measure of control or insights into powerful emotions (see Box 1).

With funding constraints ubiquitous – cost and clinically effective self-management strategies are needed to give people tools to help them self-manage their physical and psychological health, promoting the best possible long-term health.^23^ Hypnosis has the potential to help the problems associated with long-term conditions and life-limiting disease.

## The rationale for hypnosis in symptom control

Although many symptoms are generated from peripheral signals (e.g. tumour pressing on nerves) they are all processed in the central nervous system (CNS) where hypnotic phenomena are generated.^24^ Thoughts and emotions (from the cerebral cortex) as well as fear and anxiety (from the limbic system) can ameliorate or exacerbate the impact or severity of symptoms. There is a growing understanding of the concept of Bayesian brain networks as a model to explain an individual’s symptom perception.^24^ An individual’s previous experience and understanding of a symptom (‘priors’) leads to an unconscious ‘prediction’ of what their body will experience in a given situation. ‘Sensory inputs, prior experience and contextual factors’,^24^ are integrated by the CNS into the perception of the symptom; effective interventions either placebo or/and specific evidence-based pharmacological ones work through the same pathways.^25^ It is now understood that CNS has the property of neuroplasticity, that is, the pathways that generate pain and other symptoms are not hard-wired like a hi-fi but can be modulated. It is possible that hypnosis may be one way, of influencing symptom pathways, discussed in a useful review by Norman Doidge.^26^

## The evidence base for hypnosis in palliative care

There are no satisfactory trials which demonstrate the effectiveness of hypnosis in SPC. There many trials which demonstrate that it may be helpful for supportive care in cancer; for example, nausea and vomiting associated with cancer chemotherapy^27^ helping cancer pain relief,^28^ and controlling hot flushes in breast cancer.^29^ Specific evidence for EOL care is lacking.^13^ One EOL systematic review^30^ of all ‘complementary therapies’; concluded that there were no ‘satisfactory studies’ of hypnosis. A systematic review of palliative care interventions,^31^ for the National USA Consensus Project to produce clinical guidelines for palliative care, did not find strong evidence for hypnosis for any condition, though did suggest that it might be helpful in cancer pain from a study which classified hypnosis as a psychological intervention and linked it with mindfulness.^32^ This review was however, only a systematic review of systematic reviews completed since 2013, of which there were few, and none specifically on hypnosis. In addition, hypnosis was classed with mindfulness, guided visualization, and relaxation as a complementary therapy rather than as an adjunct to treatment.

Zeng and colleagues^33^ in their review of complementary therapies in hospice and palliative care patients found only two studies. Neither described the hypnotic intervention and the use of hypnosis was often blended with other approaches, making the identification of its impact alone impossible.

Other published trials include that of Brugnoli and colleagues^34^ based in a pain clinic. It was a non-randomized trial of group hypnosis – used over a 2-year period – in 50 patients to relieve severe pain (visual analog scale (VAS) score at baseline 77± baseline) in patients with cancer, rheumatological disease, and neurological conditions. They found a significant reduction in pain (measured by VAS), anxiety and consumption of opioid and other analgesics in the hypnosis group. However, the trial had many biases, for example, all interventions were delivered in the context of a fortnightly support group, there was no sample size calculation and patients chose the therapy they wished to receive.

In oncology, apart from the trials previously cited, there is a good systematic review of breast cancer care^35^ which found 13 randomized control trials (RCTs) involving 1397 participants which showed hypnosis had positive effects on pain, distress, nausea, fatigue, and hot flushes in those having curative treatment.

In summary, the low-quality evidence available in SPC allied with better evidence from supportive care in cancer suggests that hypnosis is helpful in reducing the impact of pain, distress, nausea and vomiting, hot flushes, pain, and possibly fatigue associated with cancer or its treatment. Clinicians who use it in SPC take an exploratory approach for individual symptom control.

## Clinical use of hypnosis

This description of the clinical use of hypnosis is derived from my own practice in a medical setting in palliative care and some private practice. Other descriptions can be read in standard text books such as Brann and colleagues.^5^ Some case studies (representative of people seen over a period of time rather than individuals’ exact histories) are presented in Boxes 2–4.

**Box 2.** Case history of patient with palliative and supportive care needs.

Ms CD – hot flushes during cancer treatment Mrs CD (35 years old: a single parent with a daughter of 15 years). Mrs CD was very frightened about what might happen to her daughter if she did not recover. She was troubled by hot flushes. Ms CD learned to use rapid self-hypnosis (without prolonged induction) for managing her hot flushes when they were intrusive or distressing. Her chosen imagery included (1) Ice Tap – turned on and off at will, (2) Cool sheets, (3) standing in a waterfall (most successful) Mrs CD Involved her daughter in her self-hypnosis and she found an image of a waterfall that Mrs CD used to visualize at will, feeling herself cooling off by standing under it. She also linked these cooling images to everyday activities such as turning on taps to keep the flushes at bay. Mrs CD attended in bouts – when she was having treatment and did not use self-hypnosis when she was asymptomatic

**Box 3.** Case history of someone with cancer and supportive and palliative care needs.

Mrs AB with neuropathic pain and a difficult marriage Mrs AB was about to have an operation for a spinal tumour which had presented after a short history of pins and needles and shooting pain in the legs. The day before she had received her diagnosis, her husband had told her he was leaving her. She had two children aged 8 and 10 years old. She worked in her husband’s family business and lived next door to her parents-in-law over the shop She was seen on the ward and given short session of hypnosis (with other management of symptoms and discussion of fears). Postoperatively she was seen on day 4 – she was frightened of moving (because of pain) and felt exhausted Work together included (1) reduction of anxiety and distress, (2) putting in protective structures (e.g. visors) to build up protection from emotional distress from encounters with in-laws and ex-husband, and (3) imagery for interrupting pain circuits by gates and seeing pain as images which could be managed. The pain was conceptualized as a lizard which was prevented from getting to the spinal cord by activating a special gate (4) rehearsing activities like walking or cycling in trance to help physical rehabilitation and (5) psychosocial uses of hypnosis to help re-enter and renew her social life AB was very committed and practised every day both for set sessions and intermittently when needed. She made rapid progress and was able to (1) start new self-employment, (2) separate from her husband and in-laws with reduced emotional pain, acrimony, and self-blame, (3) rehabilitate successfully, returning to riding a bike, and hiking holidays with new friends and (4) come off pain medication AB felt that hypnosis had contributed enormously to these changes particularly with the emotional pain and valued the characteristic of being able to use it at will

**Box 4.** Case history of use of hypnosis in palliative care.

Mr Z was a 66-year-old man with pleural disease and a chronic unproductive cough. He had tried oral opioids but disliked the adverse effects. He was able to use special place (a workshop). His chosen imagery involving ‘coating overactive nerve endings’ with sugary, soothing solutions. He also used imagery from his workshop skills and tools. He reduced the impact of the cough to his satisfaction after two sessions and home practice

By presenting the detail of how patients are assessed and managed for hypnosis, I hope that it will demystify the intervention and help other clinicians either to decide to train in it themselves or be prepared to recommend it.

## Is hypnosis compatible with National Health Service practice?

Many clinicians interested in using hypnosis are concerned about its possible time demands when their everyday clinical work feels pressurized already. It is perhaps easier to incorporate into palliative care where (1) clinicians have more autonomy over their use of clinical time, (2) longer individual consultations are routine, and (3) a detailed psychosocial history helpful to excellent adjunctive hypnosis is already part of standard practice.

## A personal account of using hypnosis in an acute trust palliative care team

In this section, I outline how I incorporated hypnosis into my clinical practice in an acute National Health Service (NHS) Trust, which included some home visits as part of a breathlessness service.

I did not need to extend the times of my clinics – out-patients had 1-h slots – I incorporated a 15-min hypnosis session at the end of the first consultation. Subsequent consultations were usually concerned with symptom control (including psychological distress or family complexities) and therefore hypnosis became part of the standard consultation. I also received referrals specially for hypnosis once my interest became known. I conducted hypnosis on the ward, clinic, or home. Sometimes, with permission, I carried out hypnosis in the presence of the patient’s partner/spouse or other relative. It helped the relative understand and support the use of hypnosis and sometimes learn self-hypnosis that was useful for them.

## Is your patient likely to benefit from hypnosis?

If your patient has seemingly intractable symptoms, or episodes of great distress, panic, or anxiety, they may benefit from hypnosis. People calling on clinical services repeatedly out of hours and apparently needing refuge and support rather than a clinical intervention may well benefit from hypnosis. Helping patients to acquire self-management skills (including self-hypnosis) may help individuals avoid the burden of futile admissions.

It is important to suggest hypnosis as you would any other intervention. Bear in mind that everything a clinician says at a time of heightened emotion, particularly fear or distress – will have greater resonance with the patient and family – sometimes called ‘waking suggestion’. In one sense, everything you say as a clinician is a hypnotic suggestion.

For example, using a positive explanation (without making rash promises) is likely to help increase the impact of the hypnosis if it used later, for example, ‘I believe hypnosis could be of help to you in managing the pain/hot flushes/distress that is troubling you. I have trained in using hypnosis for symptom control and it can be very helpful. Would you like me to explain more?’ A more negative approach may make the patient have less confidence – for example, ‘Hypnosis is used sometimes, I’ve no idea if it would help in your case though’. Lang and colleagues have researched the possible unhelpful effects of ‘negative language’ in pain control without the use of hypnosis, and citing this sort of research may help trainees to understand how important is the wording of interactions.^36^

Written information for the patient to consider in their own time is essential and if you are not providing the hypnosis, to be knowledgeable about the clinician who will be. If you do not know the individual you could say something like … ‘We’ve recently started to have some very good results from hypnosis as an extra help with problems like …’.

It is most important to (1) emphasize that hypnosis is not a process of handing over control to someone else, it enhances self-control^13^ and (2) that people emerge from trance even if they are not brought out of it. This is particularly important if patients are going to be encouraged to use self-hypnosis at home.

Both these statements encompass honest, positive suggestions which will contribute to the patient seeing the intervention as potentially relevant to them and reducing the sense that hypnosis is ‘other’ and outside normal clinical practice.

Young people seem particularly successful at using hypnosis – the quality of being hypnotizable is said to peak between the ages of 9 to 12 years.^37^ Hypnosis for children is not further discussed, but there are number of studies which show that it can be used to help children manage invasive procedures or investigations in cancer medicine.^13^

## What happens during a clinical hypnosis session?

In a first consultation, I would conduct an initial consultation with a special focus on the individual’s (1) personal and psychosocial situation, (2) symptoms, and (3) their priorities, and (4) previous approaches and treatments uses. I would remember any information that may give an idea what sort of imagery may be helpful for them, for example, an artist and an engineer are likely to prefer different metaphors. All these are part of a routine palliative care consultation. I would then explain hypnosis and say a little about the evidence of its helpfulness in the clinical context. I would emphasize my belief that it can be very helpful for some people, even those sceptical about it beforehand. I would also offer to send them references and website addresses with their clinic letter and give everyone our information sheet on hypnosis. An information sheet from your own institution is very useful. I would ask the person what they would hope to gain from the consultation and gauge expectations, which may need discussion. Clearly, I would never try and persuade someone into using hypnosis; sometimes people would choose to use at a subsequent consultation even if they initially turned it down. I would then focus on the information most relevant for their hypnosis session. This can be truncated, for example, for those very ill or for bedside consultations or where the first session is really to help the person achieve relaxation to relieve distress. Once severe distress has been relieved the other, more theoretical aspects of hypnosis can be covered.

## The special place and induction

The patient will create a safe/special place/mental workshop during their hypnosis sessions. They may simply use it as a safe or special place in, for example, the magnetic resonance imaging (MRI) scanner or during radiotherapy. Some sorts of radiotherapy (e.g. a mask for treatment of head and neck disease) are particularly frightening. The special place is decided by the patient either before induction in conversation with the clinician (‘waking suggestion’) or after induction. If time I would ask them to think this through in a pre-consultation phone call or letter. I would ask them to describe the place where they think they will be most calm, peaceful, and effective or the perfect place for them to spend their time. There is a huge variety of places chosen – ranging from episodes in the individual’s past (e.g. sitting by the scout camp fire when a child) to, for example, a room in their current home. One man (with a noisy family in a small house) chose sitting in the car on his drive. The place may be imaginary or even physically impossible (e.g. floating above a mountain top). Patients/clients can be given prompts to help them construct ‘the special place’ the first time, for example, what they would perceive with all their senses, what they are wearing and whether they are alone or with someone else.

I would also ask them to nominate a colour that represents fear, stress, or anger for them (or whatever their strongest negative emotion is). This is often red, black, or grey. Then I ask them to nominate a colour that represents peace, calm, fulfilment or whatever they are trying to achieve. I check whether they like stairs or prefer lifts (those frightened of lifts would not like them introduced to their induction).

Then I would find the most comfortable place available where they can sit or lie down – examination couches are not that comfortable, nor is the lighting wonderful but often that is what is available in general hospitals. I would advise any other clinic staff not to disturb us (it still happens sometimes and is not disastrous if not ideal) and then lower the lights if I can. I note the time we start and suggest that the patient closes their eyes when ready. I then ask them to start breathing in the ‘good colour’ and breathing out the bad with suggestions of feeling increasingly relaxed. I usually ask people to feel relaxation entering their body, area by area with the positive colour eventually filling it with ‘warmth and relaxation’. I would use other positive suggestions that I feel appropriate from our earlier discussion and would change the induction if needed for the second and subsequent consultations.

Then I might suggest that they breathe in and out as if on a swing, or blowing bubbles or breathing in and out like waves on the beach. I would then suggest that they are ready to go into their special place. All these stages are individualized and changed as needed. There are many other ways of inducing hypnosis.^5,14^

## Using suggestion

Once in their special place (via, stairs or lift or floating), I would start giving tailored suggestions and specific imagery based on what the patient and I have discussed beforehand or in trance (see case histories in Box 3). I use the word ‘trance’ though some other practitioners would not. There are a range of explanations and styles of clinical hypnosis.^5,14^ Many patients get helpful insights about appropriate imagery after induction.

Some ideas might be:

- Finding imagery that represents their pain and then helping them find a way to overcome it (e.g. pain circuit interrupted by a gate or barrier or diverted).
- Helping them to find a box of tools that possesses everything they need to manage their situation/difficulty confidently.
- Suggesting that they talk to their older wiser self to give advice or guidance at a difficult time – typically, this would happen in trance and the content of the conversation is between the client and their older, wiser self only.
- Difficulties can be tackled without the person having to discuss them – mental or physical equipment can be provided for them to overcome obstacles or solve problems.
- Future rehearsal is a very useful technique when a patient/client will ‘rehearse’ what they will do in an impending difficult situation, for example, receiving palliative chemotherapy and often find imagery or actions that will help them through.

## Post-hypnotic suggestions

One particular feature of using hypnosis is to implant post-hypnotic suggestions, that is, actions that the patient can take after the session has ended, linked to an everyday activity. For example, ‘every time you turn on the computer you will remember how you are re-programming your pain’, ‘every time you wash your hands you will remember how you are washing away your fear’, and so on.

Creating internal ‘thermostats’ or other instruments that can be used post-trance to adjust pain, fear, or nausea levels are frequently used.

Other imagery may be linked to the symptoms like bathing in cooling water, snow, or taps or cold wraps for hot flushes for example.

The best imagery comes from the patient and may be very imaginative.

After the work is done, I would ask the patient to open their eyes, possibly on the count of 3, 2, 1.

## How many sessions are needed and how often should they be given?

The number of sessions will be based on (1) clinical need, (2) progress with managing the symptom or problem with hypnosis, (3) the practicalities – how often the patient will/can come, (4) personal choice, and (5) clinician time constraints.

Ideally at the beginning of treatment, it is good to give people sessions reasonably close together, if they are on the ward and very unwell a daily short session is better than 1 h once a week. Out-patients can initially be seen weekly moving to monthly when there is stability. The work can be integrated with other clinical needs. Some patients only require one or two sessions in total (e.g. for chemotherapy associated nausea or hot flushes), some may need many more as they progress through treatment (e.g. with distress) or even develop new symptoms or family upset as their disease progresses.

Treatment may be given intermittently over months or years for people with chronic illness. Patients who can use hypnosis at home, are often able to come less often. I encourage people to use it regularly, as they would a drug treatment, to help it become a habit, including ‘as needed’ doses. Recordings of personalized hypnosis sessions can be provided, for example, on a mobile phone.

## Finding someone to provide clinical hypnosis

If you cannot provide hypnosis yourself, you may wish your institution to have access to this help or refer an individual patient to a clinician who can provide it.

Clinicians who wish to use hypnosis are professionally bound to receive appropriate training and use it within the scope of their practice – courses are recommended at the end of the article. There are no official qualifications regulating who can practice hypnosis outside a clinical setting, and there is a huge range of training courses available to anyone who wants to try, some only involving a short on-line training. It is essential that organizations providing hypnosis for their patients ensure that their clinicians have the appropriate training and clinical experience. Organizations like the BSCAH^8^ can provide recommendations. Clinicians who provide hypnosis assess its usefulness in the way they would any other clinical intervention and would not recommend it for everyone.

## Contraindications for hypnosis

Hypnosis is not recommended for people with delusions or hallucinations or who are abusing drugs or alcohol. People who are actively dying will not usually have the cognitive energy to engage with it except perhaps as a relaxation technique given by the therapist.

## Does hypnosis always help?

Hypnosis does not help everyone, feedback is the best way to assess its effect. You cannot judge the quality of someone’s experience of hypnosis by their appearance during treatment. ‘Time distortion’ can be a useful guide, for example, if the patient thinks they have been in trance for 5 min and it has actually been 55 that can be an indication that they probably have had a good experience. Their feelings about hypnosis, the effect on their problem and careful follow-up are the most important guides.

## The Future of Research in Hypnosis in SPC

As hypnosis imagery is so personal and has multiple complex symptoms, the norm in palliative care randomized trials are particularly difficult. Completely standardizing the intervention is not easily compatible with best clinical practice in SPC.

In some areas of medicine, such as IBS, and hot flushes, standardized imagery has been used very successfully and the use of recordings of sessions for patients to use at home.

Collaboration between SPC institutions will be important to take research forward and the impact of hypnosis itself isolated. In SPC so far, hypnosis is often tested in combination with other approaches like group work, there is heterogeneity in both methodology and in study populations and often poorly described interventions and inadequately powered trials with other significant biases.

Neuroscience is progressing rapidly and the idea of neural networks, rather than discrete areas of the brain taking on circumscribed tasks, is gaining currency.^24^ For example, meditation and mindfulness techniques are becoming understood as an activity of the ‘default mode network’. Having consensus on a neuroscientific model of the mechanism of hypnosis may help persuade other clinicians and funders of the reality of a specific impact of hypnosis. It is noticeable that academic clinicians have managed to conduct large trials of mindfulness-based stress reduction (MBSR) with widespread adoption of the practice, although it is much more time-consuming and less personalized than hypnosis, and the effect sizes reported are no more substantial (sometimes less) than those reported for individual trials in supportive cancer care.^29^

## Conclusion

There are many small trials and case reports that suggest that hypnosis could offer significant augmentation of current symptom control strategies and increasing health-building behaviours in SPC.^38,39^ There is good evidence for its use in supportive cancer care for specific situations and symptoms such as hot flushes.^29,35^ There is a documented shortfall in the psychological support that patients with cancer and palliative needs receive. There is clear evidence that this group experiences significant psychological distress, which sometimes leads to chronic depression and anxiety,^40^ which can worsen medical outcomes as well as quality of life. There is recognition that patient-initiated treatments are associated with increased self-efficacy which is known to improve medical outcomes and that this is mediated by ‘patient-centred communication’.^41^ There is frequently stated regret that hypnosis is not more widely used in medicine and surgery generally.^42^ The main barriers to wider use of this versatile therapeutic tool is the lack of robust evidence to demonstrate an advantage to hypnosis in SPC and misunderstandings about the nature of the intervention. The efforts of clinicians using hypnosis must now be to prioritize forming coalitions^5^ to pool data and then conduct clinical trials. If we do not make this change, articles on palliative care and hypnosis will continue to outline its possible benefits and yet be unable to provide supporting evidence. There would need to be agreement on the definition of hypnosis^43^ and possibly the use of use a mixed-methods approach. The work is likely to be a staged effort and will take some years to complete. In the long-term, it could lead to patients having routine access to this versatile and low-cost technique, with the potential to improve quality of life in this population who live with profound distress and, often, poorly controlled symptoms.
